# Lethal methemoglobinemia in the invasive brown treesnake after acetaminophen ingestion

**DOI:** 10.1038/s41598-019-56216-1

**Published:** 2020-01-21

**Authors:** Tom Mathies, Richard E. Mauldin

**Affiliations:** 1 United States Department of Agriculture, Animal and Plant Health Inspection Service, Wildlife Services, National Wildlife Research Center, 4101 Laporte Avenue, Fort Collins, Colorado, 80521 USA; 2 Present Address: Center for Environmental Management of Military Lands, 1490 Campus Delivery, Colorado State University, Fort Collins, Colorado 80523-1490 USA

**Keywords:** Biochemistry, Mechanism of action, Pharmacology, Ecophysiology, Invasive species

## Abstract

The invasive brown treesnake *(Boiga irregularis)* has extirpated much of Guam’s native birdlife and poses significant threats to other parts of the western Pacific. Acetaminophen (APAP) is a proven lethal oral toxicant in reptiles but the physiological mechanism is unknown. The effects of a lethal APAP oral dose on methemoglobin (MetHb, non-oxygen carrying form) levels and other blood parameters were examined in brown treesnakes. Co-oximetry was used to measure MetHb (%) and other hemoglobin species. Assessment of red blood cell integrity, white blood cell differential counts, and plasma biochemical analyses were conducted to evaluate tissue damage, stress, and liver function. Changes in oxygen carrying capacity were noted in APAP-treated snakes indicated by a 50–60% increase in methemoglobin levels and a 40% decrease in oxyhemoglobin (oxygen-carrying form) levels compared to controls. APAP-treated snakes had decreased lymphocyte and increased monocyte counts while also having increased levels of blood analytes associate with impaired liver function and muscle damage. The proximate cause of death in APAP-treated snakes was likely acute methemoglobinemia and respiratory failure due to severe hypoxia with no observed signs of distress or pain. An orally-ingested lethal dose of APAP appears to be a humane method for lethal control of this species.

## Introduction

Since recognition in the 1980’s that the introduced brown treesnake *(Boiga irregularis)* was responsible for the dramatic decline and extirpation of most of Guam’s native avifauna^1^, diverse control methods have been investigated (reviewed in ref. ^2^). Effective practical methods have been limited to those that deter entry of brown treesnakes into localized areas such as airports and cargo areas where there is risk that snakes could be accidentally transported off-island to other areas in the Western Pacific Region.

The United States Department of Agriculture’s National Wildlife Research Center (NWRC) has developed acetaminophen (N-acetyl-para-aminophenol, APAP, CAS# 103-90-2) as a brown treesnake toxicant and registered its use with the U.S. Environmental Protection Agency (EPA Reg. No. 56882-34). Unlike other existing methods of control, use of APAP-containing baits has potential for suppression of brown treesnakes on a landscape scale^3,4^. Accordingly, there is interest in the mechanism of APAP toxicosis and the humaneness of its use.

It was previously determined that an orally-delivered 80 mg dose of APAP was lethally toxic to brown treesnakes (n = 29, 47–300 g body mass) resulted in 100% mortality within 24 h (P. Savarie, NWRC, unpublished data). Similarly, Mauldin and Savarie^5^ found that 40 mg or 80 mg oral APAP doses were 100% lethal in juvenile Nile monitor lizards *(Varanus niloticus)* and Burmese pythons *(Python molurus bivittatus)*, respectively.

In vertebrates, low doses of APAP are metabolized in the liver and excreted, but ingestion of a sufficiently high dose can exceed detoxification capacity^6^. A recent study showed that several snake species cannot metabolize APAP effectively because they lack enzymes critical to the metabolism and excretion of phenolic compounds such as APAP^7^. Elevated blood APAP levels can result in liver necrosis and increased blood levels of methemoglobin (MetHB). High blood MetHB concentrations (methemoglobinemia) can be lethal because MetHb cannot bind or release oxygen^8^, resulting in death by anaemic hypoxia^9^.

In most cases of APAP-induced mortality in vertebrates, the mechanism of toxicosis is through liver necrosis and failure, but this process generally requires two to three days. Because of the relatively brief time to death observed in brown treesnakes following APAP ingestion, we hypothesized that acute methemoglobinemia was the proximate mechanism of lethality. To test this hypothesis, we performed a time-course study that characterized changes in levels of blood hemoglobin species following a lethal dose of APAP. For completeness, we examined red blood cells for abnormalities, evaluated white blood cell differential counts as indices of stress, and characterized plasma chemistry for changes in analyte levels associated with liver and muscle damage.

## Results

### CO-oximeter analysis

**Figure 1:**
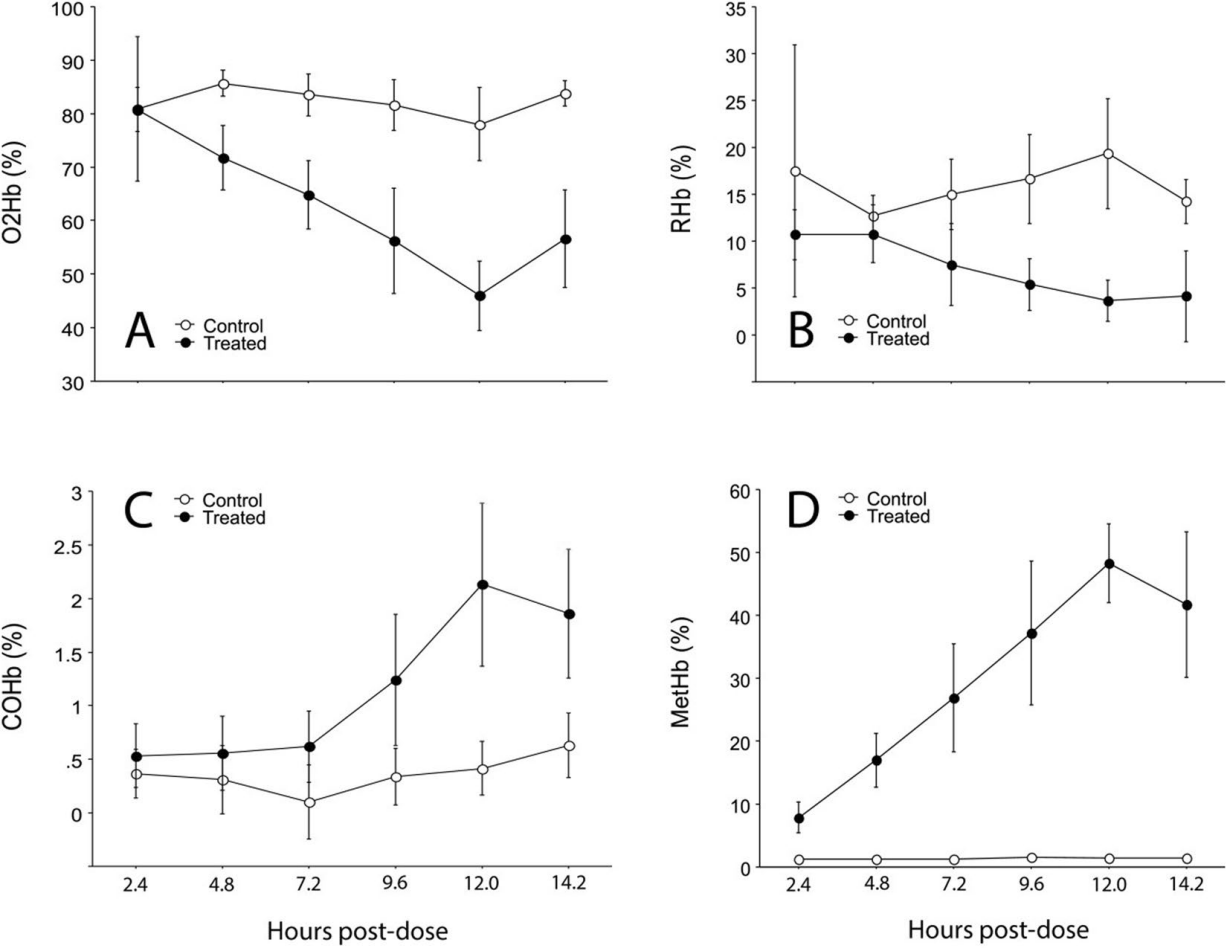
Hemoglobin levels (mean ± SD) of (**A**) oxyhemoglobin (O2Hb), (**B**) deoxyhemoglobin (RHb), (**C**) carboxyhemoglobin (COHb), and (**D**) methemoglobin(MetHb) in blood of brown treesnakes gavaged with an 80 mg tablet of acetaminophen (Treated group) or an 80 mg tablet of inert matrix (Control group) terminally sampled at 2.4 h intervals over a previously determined median time to death of 14.2 h.

The multivariate result was significant for Treatment, Timepoint, and their interaction (Table 1). The univariate *F* tests showed there was a strong effect of treatment for all dependent variables except tHb. Timepoint and Treatment × Timepoint interaction were highly significant for O2Hb and MetHb. Mean O2Hb for APAP and C groups was similar at 2.4 h post dose but declined linearly in the APAP group to a minimum at 12 h post-dose (Fig. 1A). Conversely, while mean MetHb was low and did not vary significantly with Timepoint in the C group, mean MetHb for the APAP group was higher than that for the C group 2.4 hours post-treatment, and increased linearly to a mean maximum of 50% at 12 h post-dose (Fig. 1D). Mean RHb did not vary significantly with Timepoint within either group but was always lower in the APAP group than C group (Fig. 1B). Similarly, mean COHb did not vary by Timepoint in either group, but was generally greater in the APAP group than C group, reaching a maximum at 12 h post-dose (Fig. 1C).

**Table 1:** Treatment and interaction effects for three-way MANOVA’s for hematological parameters of brown treesnakes (*Boiga irregularis*) gavaged with an 80 mg acetaminophen tablet (APAP, Treatment group) or a tablet of the inert carrier (Control group).

Hematological Parameters													
	df	O2HB		RHb		COHb		MetHb		tHb		MANOVA	
		F	P	F	P	F	P	F	P	F	P	ApproxF	P
Time	5	10.45	<0.0001	1.54	0.40	1.80	0.32	21.32	<0.0001	1.01	0.61	3.27	<0.0001
Treatment	1	127.76	<0.0001	26.64	<0.0001	17.13	<0.001	1467.50	<0.0001	2.74	0.29	369.81	<0.0001
Sex	1	0.0261	0.10	0.0003	0.99	1.94	0.39	0.34	0.71	6.19	0.06	1.93	0.10
Treatment × Time	5	7.567	0.002	2.12	0.22	0.91	0.64	20.36	<0.0001	1.31	0.49	2.76	<0.0001
Treatment × Sex	1	2.150	0.99	1.13	0.58	1.02	0.61	0.72	0.72	0.51	0.48	0.90	0.60
Time × Sex	5	0.237	0.39	0.33	0.71	0.15	0.79	1.35	0.48	1.39	0.64	0.65	0.66
Treatment × Time × Sex	5	2.933	0.055	1.16	0.58	1.00	0.61	0.71	0.72	0.27	0.99	0.94	0.55
Residual	116												

Hematological parameters are: oxyhemoglobin (O2Hb), deoxyhemoglobin (RHB), carboxyhemoglobin (COHb), methemoglogin (MetHb), and total hemoglobin (tHb). P values are adjusted to reduce false discovery rate.

There was no effect of Treatment, Timepoint, or Sex, for tHb, although mean tHb was significantly higher for females than males prior to application of false discovery rate.

**Figure 2:**
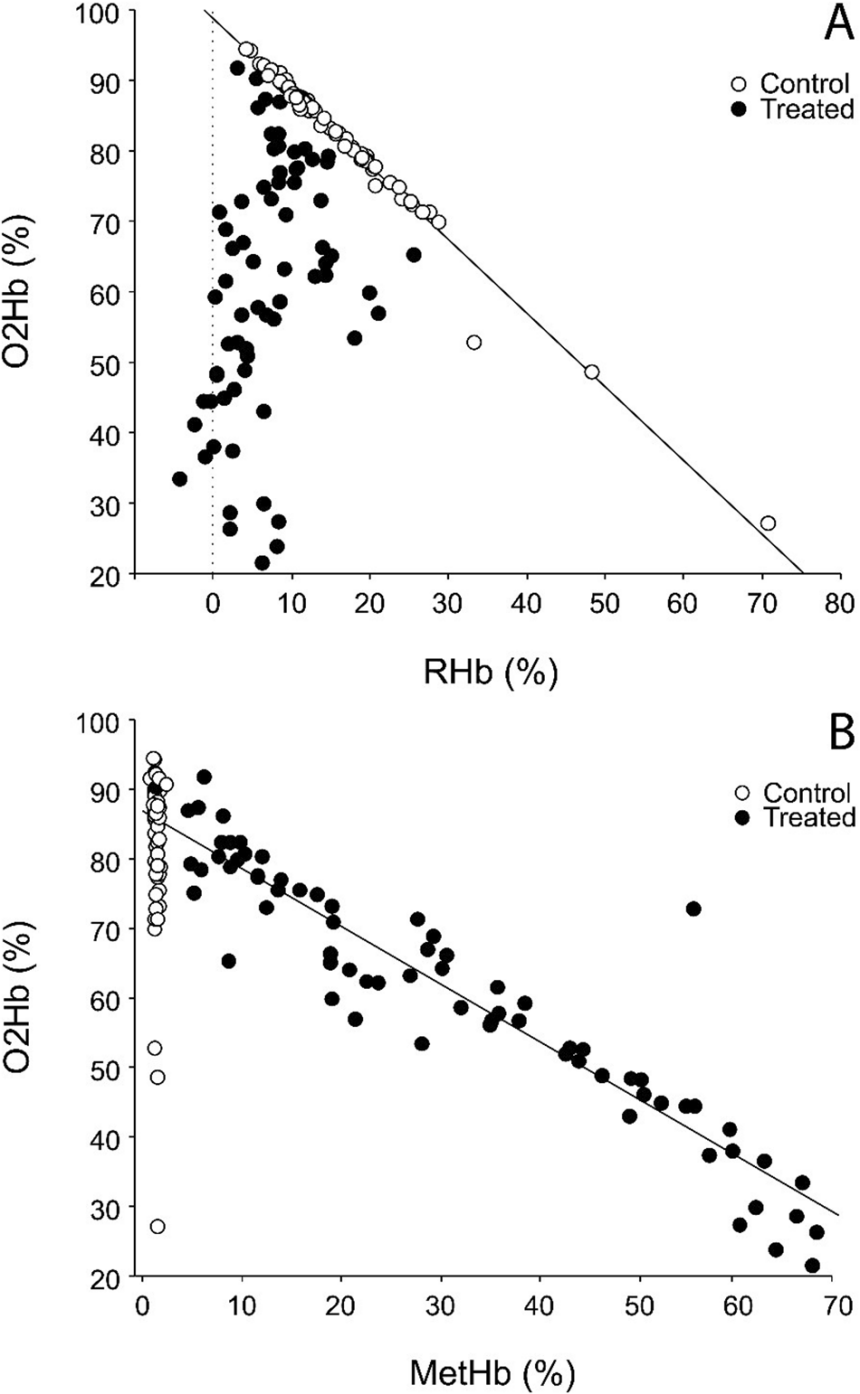
Relationship between (**A**) oxyhemoglobin (O2Hb) and deoxyhemoglobin (RHb) and (**B**) oxyhemoglobin and methemoglobin (MetHb) in blood of brown treesnakes gavaged with an 80 mg tablet of acetaminophen (Treated group) or an 80 mg tablet of inert matrix (Control group) terminally sampled at 2.4 h intervals over a previously determined median time to death of 14.2 h.

Snakes in the C group exhibited a strong inverse relationship between O2Hb and RHb (*R*^2^ = 0.98, t = 57.44, P < 0.0001; Fig. 2A) with a regression slope of −1.05 (95% bootstrapped confidence intervals: −1.09, −0.92). Conversely, there was a significant positive relationship between O2Hb and RHb in the APAP group, and the explanatory power of RHb was weak (*R*^2^ = 0.14, t = 3.38, P = 0.001; Fig. 2A). The regression slope, though also close to 1, had relatively large 95% bootstrapped confidence intervals (0.49, 1.64). The relationship between O2Hb and MetHb in the C group was not significant (*R*^2^ = 0.02, t = 1.00, P = 0.32; Fig. 2B). Conversely, O2Hb was inversely and strongly related to MetHb in the APAP group (*R*^2^ = 0.89, t = 22.42, P < 0.0001; Fig. 2B) with a slope of −0.83 (bootstrapped confidence intervals: −0.92, −0.76, Fig. 2B).

### Erythrocyte integrity

The color of freshly obtained blood in snakes in the C group was red, while blood from APAP-treated snakes ranged from dark red near the beginning of the test period to reddish brown near the end of the test period. Careful inspection of blood smear slides revealed no Heinz bodies, no untoward levels of cell lysis, or other hematological abnormalities in any of the C or APAP-treated groups, apart from one male snake in the T5 APAP-treated group that displayed pronounced polychromasia.

### White blood cell differential counts

No eosinophils were observed in the blood samples and all samples yielded thrombocyte counts of >10^3^·μl^−1^ at 12 h post-dose. The multivariate result for the analysis of WBC counts was significant for Treatment and Sex (Table 2). The univariate *F* tests revealed significant effects of Treatment for lymphocyte and monocyte counts, but not for heterophil and basophil counts. The effect of Sex was significant only for basophils, with females having higher counts than males (5.78 ± 4.64 and 2.63 ± 2.41, respectively. Mean lymphocyte count for was lower in the APAP group (25.42 ± 13.49) than the C group (50.17 ± 13.67). Mean monocyte count was higher in the APAP group (61.92 ± 13.53) than the C group (39.56 ± 13.01).

**Table 2:** Treatment and interaction effects for a two-factor multivariate analysis (MANOVA) for white blood cell types of brown treesnakes (*Boiga irregularis*) 12 h post-gavage with an 80 mg acetaminophen tablet (APAP, Treatment group) or a tablet of the inert carrier (Control group).

	df	White Blood Cell Types									
		Lymphocytes		Heterophils		Monocytes		Basophils		MANOVA	
		F	P	F	P	F	P	F	P	ApproxF	P
Treatment	1	25.32	0.0004	0.07	0.8	22.94	0.0004	0.31	0.7	7.7	4E-04
Sex	1	0.77	0.68	0.43	0.7	0.31	0.7	9.52	0.02	3.99	0.013
Treatment × Sex	1	2.58	0.29	0.74	0.68	5.25	0.09	0.15	0.77	1.67	0.19
Residual	26										

The MANOVA was followed by univariate analysis of variance (ANOVA) performed on each parameter. P values for ANOVA’s are adjusted for false discovery rate.

### Plasma biochemical analysis

Mean plasma concentrations for ALT, AST, and GLU in the APAP group (34.29 ± 14.76 U L^−1^, 38.25 ± 16.70 U L^−1^, 66.25 ± 17.88 mg dL^−1^, respectively) were significantly higher than those in the C Group (5.66 ± 1.31 U L^−1^, 12.57 ± 4.88 U L^−1^, 45.64 ± 10.16 mg dL^−1^, respectively) at 12 h post-dose (Table 3). Levels of CK differed only by sex, with males registering higher levels in the APAP-treated group (220.8 ± 66.32 U L^−1^) than the C group (66.67 ± 17.31 U L^−1^).

**Table 3:** Treatment and interaction effects for a two-factor multivariate analysis of variance (MANOVA) for plasma analytes of brown treesnakes (Boiga irregularis) obtained 12 h post-gavage with an 80 mg acetaminophen tablet (APAP, Treatment group) or a tablet of the inert carrier (Control group).

	df	Plasma Analytes									
		AST		CK		GLU		ALT		MANOVA	
		F	P	F	P	F	P	F	P	ApproxF	P
Treatment	1	28.79	0.0003	5.14	0.07	11.41	0.014	51	<0.0001	13.24	1e^−04^
Sex	1	2.067	0.2249	7.9	0.04	0.38	0.381	3.24	0.15	2.63	0.08
Treatment × Sex	1	0.248	0.6299	5.29	0.07	0.014	0.58	3.06	0.15	3.12	0.049
Residual	17										

Analytes are aspartate aminotransferase (AST), creatine kinase, (CK), glucose (GLU) and alanine aminotransferase (ALT). The MANOVA was followed by univariate analysis of variance (ANOVA) performed on each analyte. ANOVA-generated P values are adjusted for false discovery rate.

### Behavioral Observations

Most snakes remained in coiled resting positions within their hide boxes throughout the test period. The few snakes that chose to sit outside of their hide boxes also remained coiled in resting positions. A few snakes became active just prior to death, generally slowly circuiting their cage perimeter once or twice before expiring. All snakes exhibited lethargy and ataxic movement 2–3 h before death and some exhibited occasional open-mouthed gaping or yawning. Although not measured, ventilation rates of snakes in the APAP group were visibly slower 2–3 h before death than those in the C group. No snakes in either group regurgitated their tablet.

## Discussion

This is the first study to investigate the effects of a lethal dosage of APAP on blood hemoglobin species, erythrocytes, leucocytes, and blood chemistry in a reptile. Our most important finding was that the standard 80 mg dose of APAP used operationally to kill brown treesnakes results in pronounced methemoglobinemia. Elevated levels of methemoglobin were observed within 2.4 h post-dose in the APAP group (mean = 7.87% ± 4.57%) and increased linearly to a mean maximum of 50% (±SD) 12 h post-dose (median time to death = 14.4 h, see Methods). Blood obtained at this time was a dark reddish-brown color characteristic of pronounced methemoglobinemia. The elevated levels of COHb also registered in snakes in the APAP group, and although low, would have further reduced oxygen availability. In mammals, MetHb levels of 50–60% are associated with acidosis, arrhythmias, and coma, whereas death generally does not occur until levels exceed 70%^10^. Whether snakes have lower threshold to MetHb toxicity than mammals is unknown as threshold data for reptiles are unavailable. In comparison to mammals and birds, reptiles generally have lower hematocrits and lower erythrocyte counts, lower hemoglobin contents, lower blood oxygen capacities, and lower capillary densities^11^, all of which result in comparatively low delivery rates of oxygen to tissues. However, blood oxygen affinities of reptiles are lower than those of mammals^11^. Studies in fish have demonstrated that the critical thermal maximum (the body temperature at which the righting response is lost) is inversely related to methemoglobin level^12^, but it is unknown whether this relationship holds in reptiles or whether field-operative body temperatures of brown treesnakes place them at a somewhat lower threshold to methemoglobin toxicity than most mammals.

Studies on healthy reptiles conducted prior to the mid-1970’s reported levels of methemoglobin that would have been pathologic in mammals^13,14^, whereas more recent work using more reliable methods has reported much lower levels consistent with mammalian values^15,16^. Our finding for snakes in the C group (mean MetHb = 1.34 ± 0.21%) was in agreement with that of the more recent studies. Oxygenation of brown treesnake blood was variable with values of O2Hb in snakes in the C group ranging from about 70–95%, and O2Hb and RHb are tightly linked in an apparently 1:1 relationship (Fig. 2A, slope = −1.05). In APAP-treated snakes, this linkage becomes severely disrupted (Fig. 2A). In untreated snakes, there is no relationship between O2Hb and MetHb, whereas in APAP-treated snakes the relationship is strongly linear and negative. (Fig. 2B). These findings indicate that, in APAP-treated snakes, the rate that RHb is oxidized to MetHb greatly exceeds enzymatic capacity to reduce MetHb back to RHb. The continually decreasing levels of available RHb result in proportionately decreasing levels of the oxygen-carrying form of Hb, O2Hb.

Microscopic examinations of blood smears revealed no untoward degree of erythrocyte hemolysis in APAP-treated snakes relative to controls. In further support, mean tHb did not differ between the C and APAP groups. There were no indications of treatment-related renal failure (no differences between APAP and C groups in the plasma analytes CA (3.9 ± 0.0 mg dL^−1^ vs. 3.9 ± 0.0 mg dL^−1^) PHOS, (3.9 ± 1.06 mg dL^−1^ vs. 3.83 ± 1.22 mg dL^−1^), AMY (332.7 ± 152.7 U L^−1^ vs. 326 ± 157.2 U L^−1^, and K (4.35 ± 2.04 mmo dL^−1^ vs. 3.14 ± 1.03 mmo dL^−1^).

At 12 h post-dose snakes in the APAP-treated group exhibited lymphocyte counts 50% lower than counts in the C group. The effects of APAP overdose on reptilian WBC have not been investigated, but in mammals APAP induces apoptosis of peripheral blood lymphocytes^17^. It is well established that elevations in stress hormones also decrease the number of circulating lymphocytes across all vertebrate taxa^18,19^. However, increases in corticosteroid levels also increase the numbers of circulating heterophils^18,19^, and snakes in the APAP group sampled 12 h post-dose presented heterophil counts no different than those of the controls. Thus, the cause of the observed decrease in number in circulating lymphocytes, while implicating apoptosis, is unclear. At 12 h post-dose snakes in the APAP-treated group exhibited monocyte counts 57% higher than counts in the C group. In mammals, monocyte recruitment into the circulatory system is associated with inflammation^20^. The observed increase in circulating lymphocytes may have been associated inflammation of the liver^21^.

Elevated levels of three blood analytes that are routinely associated with liver damage in vertebrates were registered in snakes in the APAP group: GLU^22,23^, AST^24^, and ALT^25^. These results suggest that APAP-treated snakes experienced hepatoxicity, although in a previous study conducted at NWRC, no hepatic injury was noted on histopathological examination of liver samples collected from brown treesnakes that had died from oral administration of 80 mg APAP (P. Savarie, NWRC, unpublished data).

Treatment effect for plasma CK was significant only prior to application of false discovery rate and the observed significant Sex effect was due to elevated CK levels in males only (Table 3). In mammals, circulating CK is frequently used as an indirect indicator of muscle damage^26^ because cellular damage, death, and subsequent cell lysis result in CK leakage into the bloodstream. Muscle hypoxia can result in musculoskeletal trauma, resulting in cellular damage^27,28^. In this study, muscle damage may have been due to localized muscle hypoxia resulting from APAP-induced methemoglobinemia and a decrease of oxygen delivery to skeletal muscles. Collectively, our findings on WBC’s and plasma analytes indicate that while APAP overdose may result in limited liver and muscle damage in brown treesnakes, these effects were not the proximate cause of death in the present study.

There is an increasing movement toward the development of oral toxicants whose primary mode of action is through acute methemoglobinemia because this pathway is relatively humane^29–33^. Rapid induction of acute methemoglobinemia induces unconsciousness and death with minimal symptoms of distress. Rapidly induced hypoxia and respiratory failure is the cause of death and appears to be without appreciable pain or discomfort. Our observations on APAP-dosed snakes over the 14.4 h test period are consistent with these conclusions; all snakes in this study assumed a typical coiled resting position following dosing and most remained immobile until sampled. The few snakes that began moving about their cages did so late in the test period, were lethargic, and made no more than two circuits of their cage before expiring. Mauldin and Savarie^5^ also observed that juvenile monitor lizards and Burmese pythons lethally-dosed with APAP behaved similarly to non-intoxicated controls but became unresponsive and lethargic just prior to death while displaying no overt signs of pain or discomfort. These observations are consistent with the criteria for humane toxicant use, which include minimizing the intoxication period as much as possible, having few or no observable signs of pain or discomfort, and determining whether the physiological mechanism of intoxication is likely to cause pain or discomfort^34,35^. For further discussion of the symptoms associated with acute methemoglobinemia with respect to animal welfare concerns see Reference^36^.

## Conclusions

The proximate cause of death in brown treesnakes that ingested the standard 80 mg dose of APAP used operationally in the field for lethal control was acute methemoglobinemia and probable respiratory failure due to severe hypoxia. APAP-treated snakes exhibited no outward signs of pain or discomfort (such as writhing or convulsions), but became lethargic and unresponsive just prior to death, as has been observed in studies examining the effects of methemoglobin-inducing toxicants on other vertebrate species. The use of APAP-containing baits appears to be a relatively humane method for lethal control of brown treesnakes.

## Methods

Wild-caught brown treesnakes (n = 61 males, 78 females) were obtained from USDA Wildlife Services, Guam and held singly in well-ventilated cages in shade on racks beneath an open-air tarpaulin at WS’s facilities on Andersen Air Force Base, Guam. Each snake was fed at least one dead adult mouse after caging and was fasted for one week prior to testing. Drinking water was provided ad libitum and hide boxes were provided for refuge. Only snakes that had fed and appeared healthy were included in the study. Testing was conducted inside WS’s covered open air facilities at ambient average air temperature of 29 °C. Sex of snakes was determined when snakes were first obtained by probing for hemipenes. The study was performed under protocol QA-1302 approved by the NWRC Institutional Animal Care and Use Committee.

### Sampling design

In an initial test to determine time to death, 10 snakes were gavaged with an 80 mg APAP tablet (see ref. ^5^ for tablet production details) and the status of each individual was determined hourly until death occurred. Median time to death for these snakes was 14.4 h (range 9.8–20.4 h). Based on this finding, we performed a time course study in which six groups of snakes were terminally sampled every 2.4 h (Groups T1-T6, 2.4–14.4 hours post-dosing, see Table 4). Individuals were stratified by sex and randomly assigned to either the control (C) or APAP group, and then to one of the six sampling time points (Table 4). At the start of the experiment, each snake in the APAP group was gavaged with one tablet containing 80 mg of APAP and each snake in the C group was gavaged with a tablet containing 80 mg of inert matrix. Each snake was returned to its cage immediately after gavage.

**Table 4:** Sampling times in hr and number of animals per treatment group for brown treesnakes *(Boiga irregularis)* gavaged with an 80 mg acetaminophen tablet (APAP, Treatment group) or a tablet of the inert carrier (Control group).

Post-Treatment		Treatment			
		Control		APAP	
Sampling Time		Male	Female	Male	Female
#	hr	n			
T1	2.4	4	5	8	6
T2	4.8	7	6	5	6
T3	7.2	6	6	5	6
T4	9.6	4	6	5	5
T5	12	6	8	7	7
T6	14.4	2	9	3	8

### Behavioral observations

Following treatment, all snakes were observed for signs of behaviorally apparent discomfort and pain, including writhing, labored breathing or gasping, agitation or aggression, abnormal posture, and continuous restless movement. Resting positions and location within the cage (i.e. in or out of the hide boxes) were also noted.

### CO-oximeter analysis

At each timepoint snakes were euthanized by decapitation and pithing. Blood was collected from the carotid arteries (approximately 1 ml) into a small glass tube containing 0.025 ml of an EDTA solution and analysed immediately. Hematological analysis of blood was performed by spectrophotometry with the automated instrument IL 682 CO-oximeter (Instrumentation Laboratories, Lexington, MA) according to the manufacturer’s specifications. Hematological parameters measured were:

- O2Hb (% of total): oxyhemoglobin, oxygen-loaded form of hemoglobin
- RHb (% of total): deoxyhemoglobin, deoxygenated form of hemoglobin
- COHb (% of total): carboxyhemoglobin, carbon monoxide-bound form of hemoglobin, cannot bind oxygen
- MetHb (% of total): methemoglobin, oxidation of iron from ferrous to ferric state, cannot bind oxygen
- tHB (g/dL): total hemoglobin

### Erythrocyte integrity

An additional aliquot of whole blood from each snake was used to prepare blood smears stained with hematoxylin and eosin. These samples were examined for Heinz bodies and hemolysis using a Leica DMLS microscope (Leica Microsystems CMS GmbH, Wetzlar, Germany). MetHb is converted into irreversible hemichromes within erythrocytes that lead to the precipitation of hemoglobin in the form of Heinz bodies^37^. Heinz bodies cause lysis of erythrocytes by damaging the cell membrane^38^ and cause extravascular hemolysis when erythrocytes containing Heinz bodies are phagocytosed by macrophages^39^. These effects result in hemolytic anemia, further adding to the hypoxia-producing effects of methemoglobin.

The remaining blood sampled from each snake.was centrifuged for 3 min (3000 rpm at room temperature) and the plasma stored at −20 °C. Plasma samples were transported frozen to the NWRC in Fort Collins, Colorado, and stored at −80 °C prior to conducting biochemical analyses.

### White blood cell differential counts

Slides not immediately stained for Heinz body assessment were stored and stained later. Slides were subsequently processed using a Harleco Midas II automated stainer, with each slide dipped in a mixture of Astral Wrights stain and Harleco Wrights stain for two minutes (for color development) followed by immersion in a mixture of Wrights-Giemsa stain and hematology buffer (Astral) for four minutes. Slides were then rinsed in tap water and dried with an air blower for one minute. White blood cell (WBC) differential counts were performed on slides from the T5 C and APAP-treated snakes using an Olympus BX50 microscope, with 100 cells counted using an oil immersion lens at 1000x magnification. Heterophils, lymphocytes, monocytes, eosinophils, and basophils were reported as percentages of the 100-cell count. Thrombocyte numbers are reported as adequate or inadequate (≥10^3^ ul^−1^ or <10^3^ ul^−1^, respectively). Red blood cells were examined for polychromasia and other abnormalities.

### Biochemical analysis

Blood chemistry analysis was performed on thawed plasma from the T5 C and APAP-treated snakes using a VetScan Chemistry Analyzer (Abaxis, Union City, California) and reagent rotors containing dry or liquid reagents to quantify a variety of analytes associated with abnormal physiological states. Two rotor types containing test panels for different analytes were used. The Avian/Reptilian Profile Plus rotor contained the following test panel: aspartate aminotransferase (AST), bile acids (BA), creatine kinase (CK), uric acid (UA), glucose (GLU), phosphorous (PHOS), calcium (CA), total protein (TP), albumin (ALB), globulin (GLOB), potassium (K), and sodium (Na), while the Comprehensive Diagnostic rotor provided additional tests for alanine aminotransferase (ALT), alkaline phosphatase (ALP), and amylase (AMY). Total bilirubin was also included on the rotor but not used analytically as biliverdin is the predominant bile pigment in reptiles^40^. For analysis, each plasma sample was thawed, mixed, and approximately 0.100 ml was pipetted into the rotor which was then inserted into the analyser. The instrument performed a series of quality control tests, followed by analyte analysis and quantitative report.

### Statistical analyses

Hematological data and WBC differential counts were converted to proportional data and then logit transformed to improve normality^41^. We used three-factor multivariate analysis of variance (MANOVA) to test for the effects of the independent factors, Treatment (C, APAP), Timepoint, (T1-T6), and Sex (male, female), on the dependent hematological parameters (O2Hb, RHb, COHb, MetHb, and tHB). Two-factor MANOVA’s were used to test for the effects of the independent factors, Treatment (C, APAP), and Sex (male, female), on the dependent WBC counts (Heterophils, lymphocytes, monocytes, and basophils; no eosinophils observed) and the dependent blood plasma analytes. Only plasma analytes exhibiting observable clinically elevated levels were included in the latter MANOVA. Following MANOVA’s, we used a univariate analysis of variance (ANOVA) on each response variable to determine which dependent variables contributed to significant main effects. Because multiple ANOVA’s were conducted, values of p were corrected for false discovery rate using the “p.adjust” routine (in R), which uses an adjustment proposed by Benjamini and Yekutieli^42^.

Relationships between selected hematological parameters were evaluated using simple linear regression. Means and their standard deviations are presented as mean ± 1 SD. Differences were considered significant at P < 0.05. Statistical analyses were conducted with the R software package, version 2.15.1^43^.

### Disclaimer

Use of a trade name does not constitute endorsement by the USA government.
